# How can we make sound replication decisions?

**DOI:** 10.1073/pnas.2401236121

**Published:** 2025-01-27

**Authors:** Clintin P. Davis-Stober, Alexandra Sarafoglou, Balazs Aczel, Suyog H. Chandramouli, Timothy M. Errington, Sarahanne M. Field, Ayelet Fishbach, Juliana Freire, John P. A. Ioannidis, Klaus Oberauer, Franco Pestilli, Susanne Ressl, Daniel J. Schad, Judith ter Schure, Katya Tentori, Don van Ravenzwaaij, Joachim Vandekerckhove, Odd Erik Gundersen

**Affiliations:** ^a^Department of Psychological Sciences, University of Missouri, Columbia, MO 65211; ^b^University of Missouri Institute for Data Science and Informatics, University of Missouri, Columbia, MO 65211; ^c^Department of Psychology, Psychological Methods Unit, University of Amsterdam, Amsterdam 1001 NK, The Netherlands; ^d^Department of Affective Psychology, Institute of Psychology, Eotvos Lorand University, Budapest 1063, Hungary; ^e^Department of Information and Computer Engineering, Aalto University, Espoo 02150, Finland; ^f^Department of Psychology, Princeton University, Princeton, NJ 08544; ^g^Center for Open Science, Charlottesville, VA 22903; ^h^Pedagogical and Educational Sciences, University of Groningen, Groningen 9712 TJ, The Netherlands; ^i^Booth School of Business, University of Chicago, Chicago, IL 60637; ^j^Department of Computer Science, Tandon School of Engineering, New York University, New York, NY 10011; ^k^Center for Data Science, New York University, New York, NY 10011; ^l^Department of Medicine, Stanford University, Stanford, CA 94305; ^m^Department of Epidemiology and Population Health, Stanford University, Stanford, CA 94305; ^n^Department of Biomedical Data Science, Stanford University, Stanford, CA 94305; ^o^Department of Meta-Research Innovation Center at Stanford, Stanford University, Stanford, CA 94305; ^p^Department of Psychology, University of Zurich, Zurich 8050, Switzerland; ^q^Department of Psychology, University of Texas at Austin, Austin, TX 78712; ^r^Department of Neuroscience, University of Texas at Austin, Austin, TX 78712; ^s^Center for Learning and Memory, The University of Texas at Austin, Austin, TX 78712; ^t^Institute of Mind, Brain and Behavior, Psychology Department, Health and Medical University, Potsdam 14471, Germany; ^u^Department of Epidemiology and Data Science, Amsterdam University Medical Center, Amsterdam 1105AZ, The Netherlands; ^v^Center for Mind/Brain Sciences, University of Trento, Rovereto 38068, Italy; ^w^Department of Psychology, Psychometrics and Statistics, University of Groningen, Groningen 9712 TS, The Netherlands; ^x^Department of Cognitive Sciences, University of California, Irvine, CA 92697; ^y^Department of Statistics, University of California, Irvine, CA 92697; ^z^Department of Logic & Philosophy, University of California, Irvine, CA 92697; ^aa^Department of Computer Science, Faculty of Information Technology and Electrical Engineering, Norwegian University of Science and Technology, Trondheim 7030, Norway

**Keywords:** replication, reproducibility, methodology, reform

## Abstract

Replication and the reported crises impacting many fields of research have become a focal point for the sciences. This has led to reforms in publishing, methodological design and reporting, and increased numbers of experimental replications coordinated across many laboratories. While replication is rightly considered an indispensable tool of science, financial resources and researchers’ time are quite limited. In this perspective, we examine different values and attitudes that scientists can consider when deciding whether to replicate a finding and how. We offer a conceptual framework for assessing the usefulness of various replication tools, such as preregistration.

The ability to replicate empirical findings, accurately reproduce a data analysis pipeline, and, more generally, independently verify a scientific claim is, without question, a cornerstone of science. The aim of this dialog is not to debate whether replication is important. Our goal is to identify arguments and positions that can help us improve replication decisions, including whether a replication should be undertaken and how. The time, money, and energy required for scientific work are limited, and research groups must be judicious about where they direct their efforts.

The scientific literature, popular press, and social media are awash in reports of empirical results that do not hold up when replicated, untrustworthy results due to data manipulation and fraud, and claims of an eroding trust in science. The terms “replication crisis,” “credibility crisis,” or “crisis of confidence” are often used to describe this state of affairs, which has caused numerous fields to take hard looks at their empirical literature. These fields include, but are not limited to, medicine (e.g., ref. 1), psychology (e.g., refs. 2 and 3), economics (e.g., ref. 4), and even computer science (e.g., ref. 5). As an example from social psychology, a well-cited, large-scale replication of 100 original studies revealed that replication effect sizes were systematically lower than the original ones and that a successful replication (defined as a significant *P*-value in the replication study) was achieved in well under 50% of cases (6).

Yet, the extent and severity of these problems are contested. Fanelli (7) argues that a crisis narrative is unwarranted and counterproductive to scientific goals. He points out that in a properly working scientific field, one would not expect all reported studies to replicate, especially when one considers evolving methodology, treatment manipulations, and changes in populations over time. Consistent with this view, Shiffrin and colleagues (8) have argued that current replication issues reflect challenges that may be endemic to the practice of science, arguing that a good deal of nonreplicable results, possibly close to the present level, is necessary for science to progress optimally. However, other investigators have argued with empirical data and simulations that innovation and disruption in science has slowed down (9) despite the unilateral focus on novelty with little replication; and that discovery without replication may even have negative value if it leads to misleading waste (10) and building future work upon wrong foundations (11).

Instead of joining the discussion about the prevalence of replication issues, we will focus on how scientists can make sound replication decisions in their respective fields. We do so by examining replication through the lens of different scientific values and attitudes. In addition to describing how these values and attitudes can guide replication decisions, we examine how different replication tools, such as hypothesis/model preregistration, large-scale collaboration efforts, and various journal reforms, can be aligned, or not aligned, with them. Consistent with the stated goals of the special feature “Dialogs on the Practice of Science,” (12) we do not provide rigid recommendations on the practice of science. We offer differing perspectives and note that replication challenges are not likely to be solved with one-size-fits all scientific reform. We hope that our dialog is useful for guiding future, field-specific discussions and debates on replication practices.

## Epistemic and Nonepistemic Values

Building on the work of McMullin (13), and others (14–16), we distinguish between epistemic values and nonepistemic values in science. Epistemic values provide valid reasons for thinking that a hypothesis or scientific statement is true or not (13). For example, one could consider the observed predictive accuracy of a model as an epistemic value (17). Nonepistemic values, in contrast, can influence scientific decisions and actions but do not directly relate to the truth of a hypothesis or scientific statement. Nonepistemic values can include the ethics of research activities, policies that could be enacted based on the outcome of a study, and even personal or religious beliefs.

Epistemic and nonepistemic values can jointly influence scientific decisions, in both positive and negative ways (18), for example, suppose we were carrying out a vaccine efficacy trial, where the vaccine carries the possibility of serious side effects. The nonepistemic value of mitigating harm to the larger population of individuals who would be receiving the vaccine if it were deployed at scale could be considered when determining the sample size of the trial or the setting of statistical thresholds for claiming efficacy. The idea would be to ensure that the study yields definitive results before the vaccine goes into mass production, thus protecting the population being vaccinated. Epistemic values, such as the careful evaluation of the subsequent statistical modeling, would guide how we determine whether the study was successful (e.g., refs. 15, 17, and 18). For an example with decidedly negative consequences, suppose a researcher allowed the nonepistemic value of increasing one’s fame or clout to override epistemic values relating to the fair evaluation of hypotheses after collecting data. This conflict of values could lead to data fraud and data manipulation.

## Two Simple Cognitive Attitudes

Elliott and Willmes (17) have argued that the cognitive attitudes of scientists play a major role in determining how values, both epistemic and nonepistemic, are weighed when making scientific decisions. They define a cognitive attitude as a scientist’s evaluative response to a claim, hypothesis, model, or theory (17). For example, a scientist may choose to outright believe the claim that people can hold seven, plus or minus two, pieces of information in their short-term memory at any given time.^*^ Others may consider it roughly true with caveats. Others may not believe it to be true per se, but consider it a useful hypothesis to argue against. Whether, and how, a scientist chooses to replicate a particular memory study will depend upon their cognitive attitude regarding this claim.

We present two cognitive attitudes to aid in our replication discussion that are grossly oversimplified, almost to the point of caricature, but are useful in drawing out competing perspectives on replication. These cognitive attitudes center upon how a scientist evaluates claims within the peer-reviewed literature and reflect what normative role the scientist thinks the peer-reviewed literature should serve and how it should be used.

The first we term the Book of Truths cognitive attitude. Someone who holds the Book of Truths attitude believes that claims within the peer-reviewed literature are, or at least should be, a collection of truths, or facts,^†^ and all efforts should be directed toward making it so. If an empirical result fails to replicate, the original claim should be corrected or retracted. What if all researchers held the Book of Truths attitude? No effort or expense would be spared on direct replications and large-scale many-labs collaborative experiments would be the norm. The resulting peer-reviewed literature would be straightforward to use by nonexperts, such as policy writers, politicians, and journalists, as most results could be accepted at face value.

At another extreme, we consider the Book of Conversations attitude. Someone who holds the Book of Conversations attitude believes that the peer-reviewed literature is, or at least should be, nothing more than a method of communication, and an exchange of ideas, among scientists, i.e., a conversation. Claims are to be considered carefully, but not necessarily believed to be true, or, at least entirely true. There is an acknowledgment that results may or may not replicate; the focus is not on the truth of statements but rather that the literature is an accurate and detailed record of what was done. What if all researchers held the Book of Conversations attitude? It would not be a priority to directly replicate all empirical phenomena nor would there be an expectation to do so. Different papers examining the same empirical phenomenon would likely use different experimental protocols and designs, leading to a richer, more diverse “garden” of findings and methods. All else equal, this could result in a greater rate of discovery, but might come at a cost of inefficiency when results are inconsistent. More time and effort would be directed to other research activities than direct replications under this attitude. It would be difficult for nonexperts to read and apply the scientific literature, as assessing the robustness and validity of findings would require, at the very least, field-specific expertise.

**Fig. 1.**
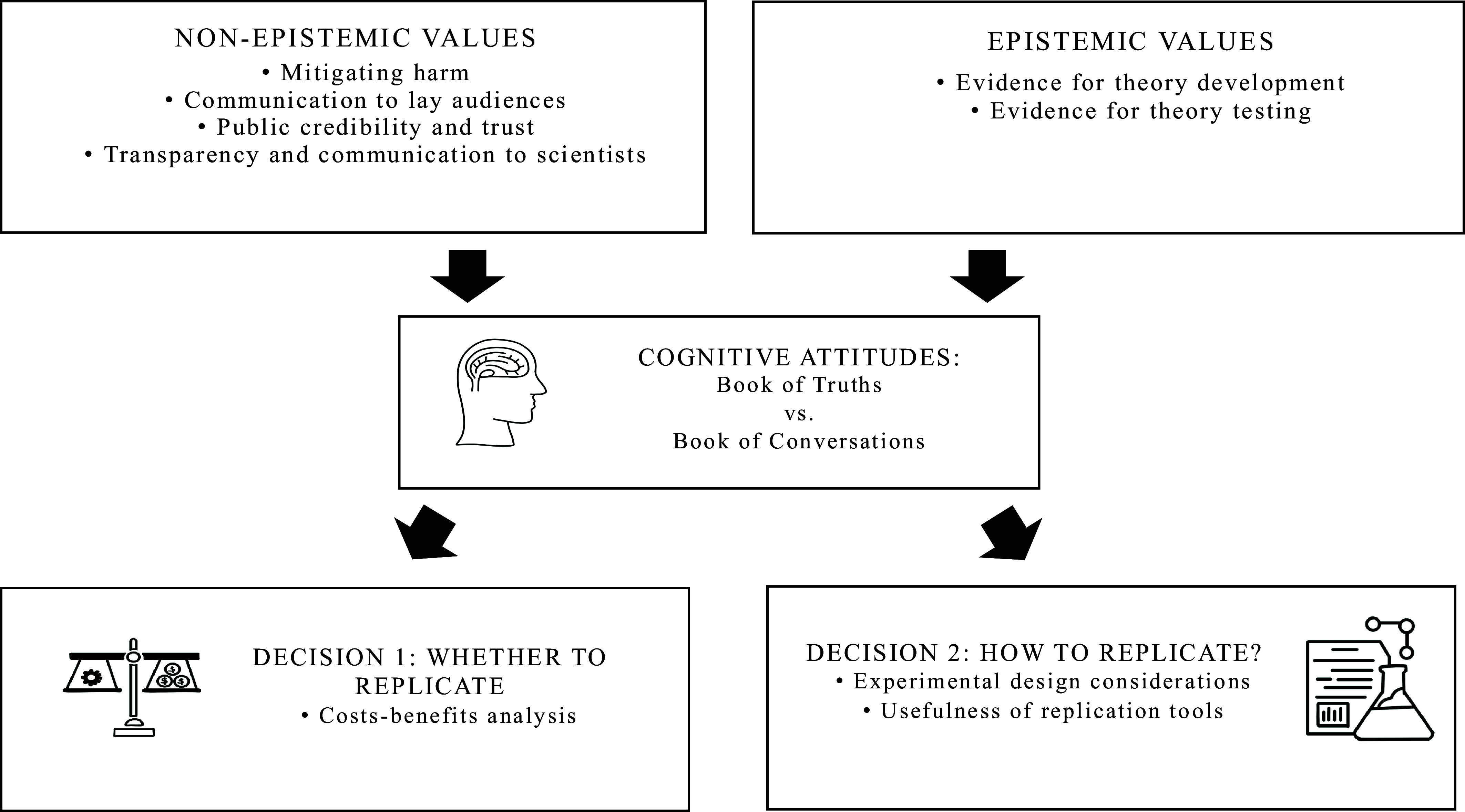
This is an illustration of the conceptual framework we used to guide our discussion. Values, both nonepistemic and epistemic, influence decisions about replication, which are, in turn, weighed via the cognitive attitudes of the scientist making those decisions.

Fig. 1 provides an illustration of the conceptual model that we used to guide our discussions. Replication decisions are treated as the outcome of a deliberative process where epistemic and nonepistemic values are evaluated through the lens of our two cognitive attitudes. We argue that some values are better aligned with one cognitive attitude than the other, which has practical implications for replication decisions. In our discussions, the confluence of values and attitudes impacts both the decision to replicate as well as how to replicate, see Fig. 1. Going a step further, considering replication decisions in this way allows us to better understand when different replication tools, such as registered reports and many-labs experimental designs, will be effective. It is far beyond the scope of this discussion to provide a complete accounting of all relevant values, so we chose to focus on a few salient ones.

## Nonepistemic Values Relating to Replication

### Mitigating Harm.

Returning to our previous example, consider the development of a new vaccine that is to be deployed to billions of people. Different labs from across the world have identified several promising vaccine candidates. As with any vaccine, there will be questions of efficacy as well as—potentially harmful—side effects, which will depend upon the nature of the disease and individual health factors (age, preexisting conditions, etc.). Suppose that initial trials for a candidate vaccine appear to be quite promising, showing an immune response with minimal harm. Under emergency statutes, these initial trials follow an adaptive design (22, 23) based on relatively small samples (usually of healthy volunteers). After these initial trials, crucial questions about candidate vaccine safety and efficacy remain.

This example nicely illustrates the nonepistemic value of mitigating harm. How should we consider replications of the empirical clinical trials? Obtaining consistent, accurate results in these empirical replications would be the highest priority. If the results are inconsistent, or inaccurate, the attribution of serious adverse events could be misinformed, leading to real harms for many individuals and eventually an unfavorable benefit–risk ratio. When adjudicating among multiple candidate vaccines, unreliable estimates (e.g., about efficacy and/or harms) could cause an inferior vaccine to be mass-produced. Further, conflicting reports in the scientific literature could erode trust in the population intended to receive the vaccine, complicating deployment and uptake. For these very reasons, a Book of Truths attitude would be well-aligned for a scientist to have in this case. The planned replication studies need to provide a definitive result, at the level of a fact, if that is at all possible, and the literature itself needs to reflect this clarity.

For this example, ensuring accurate results would require considerable investments of money and time. Depending upon the efficacy of the drug and/or diversity of the target population, it may require studying many participants or patients, from different demographics and possibly different cultural backgrounds, to achieve the required accuracy and certainty that benefits far outweigh the harms. If the potential harm is serious and/or common enough, then it is worthwhile to spend the resources.

Effective replication tools in this regard are large-scale replications, which coordinate efforts across multiple sites. In the medical field, randomized controlled trial data can be collected in large, multicenter collaborations that are essentially direct replications per center (*many labs*). More recently, such collaborations are put in place to study more than one research question in platform trials (e.g., RECOVERY in COVID-19, and STAMPEDE in prostate cancer). The goal of the participating centers is not necessarily to refute or contextualize the results from a different center, but to increase the precision of the estimates such that valid conclusions can be drawn. Apart from these top–down collaborations, bottom–up collaborations have increased in popularity where multiple randomized controlled trials are designed separately but similarly (close to exact replications) and can be jointly analyzed in a prospective meta-analysis (24–26). Top–down collaborations are usually funded in advance, and therefore limited to a certain sample size. Bottom–up collaborations have decentralized funding and can easily inspire new research teams with new funding to join the effort. These replications mitigate harm by increasing the sample size and precision of the estimates, usually with the goal of reaching a conclusion earlier (to impact patient care as early as possible), or to not let the sacrifice of the patients in the trial go to waste in an inconclusive result.^‡^

### Communication to Lay Audiences.

There is undeniable value in expanding access to discoveries (27), promoting education (28–30), and engaging the general public in research activities (31). This brings forth the challenge of effectively communicating science to nonexperts. The scientific literature is read by a wide range of people, including politicians, policymakers, journalists, and concerned individuals with a personal stake in the science, such as health concerns.

Scientific findings are often communicated as definitive and exacting when, in reality, science is uncertain, iterative, and messy. The misunderstanding of scientific concepts and empirical results is, unfortunately, quite common, especially when involving inherently probabilistic information (32–34). This can negatively influence laypeople’s attitudes and real-life decisions, such as those related to vaccination (35). We can think about the cause of misunderstanding as a mismatch between cognitive attitudes. It is problematic if a lay audience member, say a journalist, reports on a published study assuming a Book of Truths attitude when, in fact, the available evidence is mixed and the original authors themselves viewed the study more from a Book of Conversations attitude. This mismatch might lead the journalist to overclaim a study’s result when the intent of the study was not to be a definitive statement on the phenomenon in question, but, rather, an exploration of different experimental manipulations that contributes to the scientific conversation on the topic. As such, failures to replicate can be perceived as undermining the role of science as a reliable producer of knowledge instead of appreciating that replication is a mechanism of understanding uncertainty and scientific progress (36). It should be noted, nevertheless, that the problem often originates from scientists, their institutions, and their public communication channels that overhype results and circulate press releases that make extreme claims (37–40).

Replication can help address some of these issues. In the short term, it enables a prompt and comprehensive examination of new findings, thereby enhancing their reliability, and minimizing the risk of errors (41). In this context, prioritizing the replication of results that are immediately relevant to the public, for example, due to urgent requests or the influence it will have on policies, especially if these results are highly “surprising,” could be crucial, given their potential to impact a large number of individuals. To consider a concrete example, one may wonder whether a quicker disproof of the fraudulent claim regarding a causal link between vaccines and autism may have alleviated vaccine hesitancy and the conspiracy theories that fueled it for decades. More generally, the negative consequences of public policy built upon poor, or grossly incomplete, research also relates to the nonepistemic value of mitigating harm. Replicability might also contribute to clarifying the conditions under which an effect holds (42), thereby better informing the actions of institutions responsible for translating scientific findings into practical regulations.

Communicating the nuances of replications to nonexpert audiences could be improved by the implementation of new reporting practices within peer-reviewed journals. For example, in addition to the summary of results directly usable by the general public, the level of support for the author’s conclusions based on their data could be reported, with an emphasis on contextualizing this strength of evidence with prior studies, preferably in a systematic review (10). One could also provide more detailed limitation sections in the paper and include this information in the abstract. This is particularly relevant for studies that will inform decisions that have the potential to impact a large number of individuals, such as policy decisions (e.g., refs. 43 and 44). Also, linking replications to the original research (45) could help audiences to understand whether the results are disputed, and this, in the long run, may help educate on the overall progress of science. Improving the science literacy of lay audiences is an ambitious task but could enable more informed individual and collective decision-making (46, 47).

Could the scientific publication system accommodate both a “Book of Conversations” and a “Book of Truths” attitude? This could be accomplished by an explicit, clearly visible distinction between publications that serve the goal to communicate new findings and ideas to other scientists, and publications that are intended to report a finding or conclusion that is more firmly established and ready for public consumption. For instance, Lewandowsky and Oberauer (48) proposed that original studies are published as “provisional” before having been replicated, with an invitation to other researchers to replicate it. After successful replication, the report is promoted to archival status, including the replicators as coauthors. After a failure to replicate, the publication is withdrawn and replaced by a public record of the replication failure. These records aim to reduce potential public interest and simultaneously enable reuse, for instance, by later meta-analyses. Although, as we later discuss in this dialog, it can be a highly nontrivial task to define what constitutes a successful (or failed) replication attempt. A version of this proposal is implemented by the *Journal of Artificial Intelligence Research* (45), where instead of replacing original studies that are not reproduced by a record of replication failure, articles that are reproduced receive a badge that states this explicitly. Also, reports describing the replications are published alongside the original articles, whether they replicated or not, to better contextualize the effects being investigated and allow for data reuse (45). Such a differentiated publication system would make the different levels of evidence for published findings more transparent for lay audiences and could help communicate to the public how science works.

### Public Credibility and Trust.

Public credibility and trust is a value worth considering when making replication decisions. Acting responsibly as researchers—which involves, among other things, conducting original research with transparency, honesty, and accountability—plays a crucial role in the public’s perception of our credibility as a community. It follows, then, that decisions regarding replications, should be made responsibly, with an eye to continuing to earn (or, for some areas, to earn back, in the wake of replication concerns, see refs. 6, 49, and 50) and maintain the public’s trust. Increasing public credibility naturally aligns with a Book of Truths attitude, with its focus on establishing repeatable findings, which helps establish public trust. A person holding the Book of Conversations attitude is less concerned with what the public finds credible, as the literature is viewed primarily as a conversation among scientists themselves (insiders). This can become problematic if the knowledge gained from studies is not clearly conveyed to the appropriate audiences.

One useful tool that can help improve public credibility is the registered replication report format (51). When replication studies are conducted as a registered report, the plans for the study, including sampling and analysis strategies, are registered and peer-reviewed before the study is conducted. Although the registered replication report format does not guarantee this, it can improve the likelihood that a replication study will be methodologically sound, and that researcher degrees of freedom and bias will be limited. When coupled with prospective meta-analysis designs, this can yield more accurate estimates of effect sizes, which can help combat the “winner’s curse,” where initial studies report inflated effect sizes, only to have those effect size estimates dramatically lowered upon replication (52). More consistent, accurate effect size estimates can help build credibility and trust with the public.

### Transparency and Communication to Scientists.

Transparency and communication with other scientists is a nonepistemic value that aligns with both of our cognitive attitudes, but for different reasons. From a Book of Truths perspective, transparency enables direct replication by providing scientists with all necessary experimental details. From a Book of Conversations attitude, transparency and communication to other scientists is the primary role of the peer-reviewed literature.

Providing transparency and accurately communicating all relevant aspects of a study is integral for replication efforts, even more so when real benefits and harms are at stake. The Reproducibility Project: Cancer Biology (53–55) demonstrated major difficulties in replicating preclinical research. A contributing factor to this was that methods were insufficiently documented for the majority of studies, and they simply could not be completed. Additionally, reanalyses in the biomedical sciences have shown failed outcome reproducibility (i.e., the original results could not be confirmed by rerunning the original analysis scripts on the original data) and occasionally major differences between published and reanalyzed results for pivotal commercial trials (56), likely due to insufficient transparency in analysis plans and methods.

There are two main components of replication to consider here. The first is to be able to recreate the study itself and successfully rerun all the analyses. This requires not only an adequate, detailed description of the methods, but also the sharing of the raw data and of the specific code used in any and all reported analyses. The second component is to be able to use the presented information on methods, including all the processes undertaken and the analyses conducted, in order to design one or more new studies that use the same methods, processes, and analytical pathways. Regardless, the ability to meaningfully perform exact, similar, or diverse replications depends on the ability to understand what happened in the original study.

Notions of transparency and reproducibility can lie on a continuum. Within the field of computational science, Peng (57) describes a “reproducibility spectrum” where, at one end, the publication contains no code at all, and at the other, everything is provided to fully reproduce the results, which includes all of the code, data, linked executables and dependencies, etc. The reproducibility study type can be distinguished based on which documentation it relies on, such as the article describing the original study, code, and data, while the degree to which the study is reproduced can be characterized by whether the result obtained is exactly the same, different but leads to the same conclusions, or if an alternative analysis leads to the same conclusion (58). All else equal, the more data and code that are available and easy to use, the better for reproducibility purposes (59). Data and code sharing is also indispensable for evaluating new models under alternative specifications.

Yet, making results fully reproducible^§^ is not without costs. Within the field of computer science, making computational experiments available can be as easy as creating a Docker container (60) (i.e., an executable package, including the code, system tools, dependencies, and settings necessary to run the experiment), but producing quality software that is open-source, fully documented, and can be reused and extended is a time-consuming and costly endeavor. It is not only time-consuming to produce code, the hours spent maintaining it and answering questions regarding documentation can prevent the scientists from working on new research. Costs in the form of money could also be huge. Studying the emergent behaviors of large language models (61) requires access to the largest models, which currently are the commercial ones and cost more than 100 million United States dollars to train. It is worth mentioning that these commercial models are neither open nor transparent.

In light of these potential costs, one perspective that scientists can take is that “natural selection” will determine which results are important enough to warrant the additional costs required for full reproducibility. Using computer science as an example, if a computational method is shown to be effective, naturally, improved versions of the code will be developed and released, typically by other labs. On the flip side, requiring this effort for the initial publication may hinder scientific progress, in particular, for the long tail of scientists with limited resources. The idea is to require transparency in what was done, but not necessarily full reproducibility. This perspective aligns well with a Book of Conversations attitude, as the literature is viewed as a transparent exchange of promising ideas among scientists, with the understanding that not all research results are expected to be fully reproducible at face value.

Moving the computer science literature in a direction where all computational products are available and fully reproducible would require considerable time and effort. Consider, for example, the Association for Computing Machinery Special Interest Group on Management of Data Conference Reproducibility Evaluation effort in which authors of accepted papers were invited to submit their computational experiments for evaluation of whether all results (e.g., figures, tables) in the paper could be easily reproduced from the code and data that were made available. Over the past 15 y of this program, only around 25 to 30% of the authors submitted their experiments for evaluation. In contrast, by lowering the bar and requesting authors to just make their code and data available, over 65% of the accepted papers made their artifacts available at Very Large Data Bases Endowment 2021 (62). A similar effort at the NeurIPS conference increased code sharing from below 50% to nearly 75% in one year (63). Making the code available, though, might not be enough, as a study found that less than 50% of code was executable even after communicating with the original authors (64).

Regardless of how we view these reform policies, there are now many tools, platforms, and repositories that can help in methodological documentation and transparency. For example, in the biological and biomedical sciences, decades of commitment have led to major advances in reproducibility practices. This commitment has spurred a multifaceted effort to improve methods and infrastructure and encompass reporting guidelines (e.g., refs. 65, 66, 67), or highly standardized and specialized data repositories (e.g., ref. 68).

## Epistemic Values Relating to Replication

### Evidence for Theory Development.

Theory development is the endeavor by scientists to find explanations for empirical phenomena—empirical regularities that can be observed repeatedly across time and situations. By “explanation,” we usually mean some set of theoretical assumptions that, if true, render the phenomena in question substantially more likely than if they were not true. To be worth explaining, a phenomenon must not only be stable over time—as demonstrable by direct replication under nearly identical conditions—but also general across many situations (69), and robust across several different methods for observing it (70). For example, theories of memory aim to explain the shape of the forgetting curve (71) because it is observed across many kinds of memory contents, types of memory tests, and person populations. A theorist aiming to find an explanation for a phenomenon needs to know as much as possible about its scope of generality because a candidate explanation needs a corresponding scope. It follows that, to advance theory development, empirical research needs to prioritize establishing the generality of an empirical regularity through conceptual replication as much as establishing its robustness through direct replication (69, 72).

The epistemic concerns that relate to the development of theory derive in large part from those associated with the underlying phenomena—a theoretical framework is not made better if it explains phenomena that are fragile to variations in the experimental design or study sample. From this perspective, a Book of Truths attitude more naturally aligns here as a theory is difficult to develop if one does not know for certain what phenomena to explain. Publication bias presents a major threat to establishing phenomena and its boundary conditions. The occurrence of publication bias may affect theory development in two ways: first in the establishment of explananda, and second, in the testing of new predictions against the empirical literature. If a theory predicts a phenomenon not usually considered in a given context, and experiments so far have failed to detect that phenomenon, but publication of these null results have been suppressed, then false theories may remain in the literature unchallenged; it may even be buttressed when false positive results occur later. Replication tools intended to limit or eliminate publication bias naturally align here, such as registered replication reports (51).

However, Feest (73) challenges the usefulness of direct and conceptual replications by arguing that they offer very limited information about the nature of an effect to be explained. She argues that for research paradigms that are of limited “conceptual scope,” that is, the causal relationships between the independent and dependent variables are not well understood, it becomes nearly impossible to know what variables to hold constant (for a direct replication) or systematically manipulate (for a conceptual replication)—see also refs. 74 and 75. The crux of the problem is that if we carry out a replication of either kind and observe the “same” effect, we have no idea whether it truly is the same effect being observed or whether it is something completely different. We often do not know which experimental variables are core to the effect and which are auxiliary. In other words, the effect of an independent variable on a dependent variable is often confounded by interactions with background factors, either held constant in the replication study or varied in ways not accounted for by the researchers, leading to aggregation fallacies (e.g., ref. 76). Feest illustrates her points with the “Mozart effect”—a result first reported by Rauscher at al. (77) whereby listening to a Mozart sonata temporarily improved participants’ scores on a spatial reasoning test. A considerable amount of subsequent empirical work identified that: i) this temporary improvement was much smaller than originally identified (78) and ii) the effect was attributable to the arousal in mood one may experience when listening to upbeat music, but the stimulus need not be musical at all. Both the sonata, specifically, and the music itself, more generally, played an auxiliary role to the effect.^¶^

Feest (73) proposes to solve this problem by considering experiments designed to evaluate effects not as replications per se, but as systematic “explorations” of the variable space. This perspective better aligns with a Book of Conversations attitude as the focus shifts from establishing the “truth” of an effect via direct replication to building out a series of results that systematically inform us about the behavior of various dependent and independent variables, a notion echoed by ref. 80. One could debate whether this is conceptual replication in a different guise, but the intent and subsequent interpretation are perhaps different (for thoughtful discussions, see also refs. 81–86). While compatible with multiple values and attitudes, metastudies (87), which systematically randomize the values of multiple independent variables when designing experiments, are particularly well-suited here. Modeling frameworks that help delineate the logical structure of replication experiments would also be beneficial (88).

### Evidence for Theory Testing.

We need to ask which study promises a larger gain of information for our research question when prioritizing between two empirical studies that have about the same cost. If the aim is to establish a phenomenon, we should ask how much information we gain by corroborating or debunking the hypothesis that it is real. On the other hand, if the aim is to test a theory, we should instead gauge the information gained from a study of the credibility of the theory. The two aims can lead to different evaluations. For establishing a phenomenon, the choice is between a direct replication of a first study supporting the hypothesis, or a conceptual replication assessing its generality across some dimensions. For theory testing, the choices are different. A good theory predicts not only a single phenomenon but several phenomena, and therefore offers multiple avenues for testing the theory. After researchers have run a first study yielding support for one prediction of the theory of interest, a second study testing another prediction of the theory often promises a larger information gain than a direct or a conceptual replication of the first study (72). However, see Davis-Stober and Regenwetter (89) and Heck (90) for an argument regarding the inherent challenges of interpreting evidence aggregated across studies that each test different predictions of a theory.

Theories are typically grounded in multiple sources of evidence and are intended to account for various phenomena. Therefore, the direct replication of a single experiment, even if successful, does not necessarily offer strong support for the overall theory. Converging evidence obtained through different methodologies, and sometimes by different research teams (i.e., triangulation), may be more informative, especially when considering different predictions of the theory. Specifically, if diverse pieces of evidence confirm various predictions of the theory, the theory is strongly supported. The more diverse these pieces of evidence are—either with regard to their content or the methodology that has produced them—the stronger the support for the theory when they converge. Even unsuccessful replications can be informative because they might contribute to a better understanding of the theory. However, one should worry that allegiance and publication biases may generate a literature of reported scientific findings that all seem to support a theory, but the entire literature may be spurious. Even the most squarely refuted findings continue sometimes to be heavily cited, often without attention paid to the refutations or with excuses from the supporters of the theory who are unwilling to let go (91, 92).

The aim of testing a theory has implications for the role of preregistration. Oberauer and Lewandowsky (72) distinguish between preregistration of hypotheses and of analysis plans, which serve different aims. Preregistration of hypotheses is advocated as a means to distinguish between a priori predictions and post hoc interpretations of findings. When a study aims to test a strong theory that unambiguously predicts a particular outcome, then the theory itself already serves the role of preregistering the hypotheses. In the best case, the theory is a formal model that we can run to compute the predictions for a study. With strong theories, the preregistration document merely serves to document the hypotheses that follow from the theories. When the theory to be tested is weak, so that what it predicts depends strongly on rather arbitrary auxiliary assumptions, then preregistration can serve to make the auxiliary assumptions explicit. Preregistering such assumptions, however, does not make them less arbitrary. Therefore, in the case of weak theories, empirical confirmation of preregistered hypotheses does not provide more support for the theory than confirmation of an alternative, not preregistered hypothesis that could be derived from the theory with different, equally arbitrary auxiliary assumptions. Hence, preregistration of hypotheses is a good opportunity to make theoretical assumptions explicit, but it adds nothing to the conclusions we can draw from the results of a study for the theories we aim to test by it (72).

A second aspect of preregistration is to commit to an analysis plan before seeing the data. This serves to reign in problematic research practices such as *p*-hacking, where many analysis paths are tested but only those that show support for a theory, or are otherwise desirable for the researchers, are reported in a paper (93). Usually, showing an effect is more informative, and more publishable, than not showing it, and therefore, *p*-hacking is likely to artificially inflate the size of reported effects. Preregistration of an analysis path may help to reduce researchers’ freedom to report cherry-picked results from a large number of analysis paths. Evidence that this is effective comes from a study of published effect sizes in psychology (94), which showed that studies with preregistration had considerably smaller effect sizes than studies without preregistration.

For the aim of theory testing, preregistration is probably most effective in the form of a registered report: Study design and method, hypotheses to be tested, and analysis plan are not only registered publicly but submitted for peer review. After passing review, the publication outlet commits to publishing the study regardless of whether the outcome supports or challenges the theory. This provides an opportunity for proponents and opponents of the tested theory to agree on reasonable auxiliary assumptions for deriving predictions, on a diagnostic design, and adequate analysis methods. Such a negotiation reduces not only the freedom of the research team to choose the auxiliary assumptions and analytical paths that suit their goals but also the freedom of their opponents to dismiss the evidence. In some areas of medicine, such as clinical trials, there are already many thousands of protocols that have been published upfront in peer-reviewed journals and there are also large numbers of detailed statistical analysis plans that are published upfront before a trial is run (95–98). Yet, even with detailed prespecified analysis plans, deviations are very common (99).

## When Is a Replication Successful?

Our discussion has largely focused on decisions regarding the design, motivation, and communication of replication studies. We have not yet considered how to decide if a given replication is successful. To engage with this question, at even a superficial level, we require a definition of replication success. Given two studies, each of which have obtained their own data and are intended to answer the same question, a successful replication is defined as follows by the *Committee on Reproducibility and Replicability in Science* from the National Academies of Science, Engineering and Medicine.

- Two studies may be considered to have replicated if they obtain consistent results given the level of uncertainty inherent in the system under study (100).

Determining whether a pair of studies produced “consistent results” can depend upon the application of statistical methodology. Many researchers have argued that an overreliance on null hypothesis significance testing (NHST) has contributed to replication problems across many disciplines (e.g., refs. 101–103). One of the biggest criticisms of using NHST for defining replication success is that it dichotomizes results—success or failure—in ways that lead to biased reporting and fallacious reasoning. For example, an effect may be present in a population, but, due to natural sampling variability, some replications may be statistically significant by NHST [typically according to an arbitrary threshold of evidence (104)], while others are not. This leads to a published literature with upwardly biased effect size estimates when editorial decisions are based on achieving statistical significance (e.g., ref. 21).^#^ See refs. 101, and 105–108 for additional examples and discussions of how dichotomous reasoning using NHST can lead to fallacious decision-making, especially within a replication context.

The development and interpretation of statistical methods for assessing replication is an active area of research, with many promising avenues (21, 109–112). A full accounting of modern approaches is beyond the scope of our discussion, but some guiding principles include 1) taking an estimation perspective, i.e., prioritizing interval estimates, and considering evidence in a continuous fashion (21), 2) leveraging Bayesian decision-making (109–111), and 3) moving beyond goodness-of-fit indices (112). There are also recent methods designed to estimate and characterize heterogeneous treatment effects (113). We do want to highlight that determining whether a study replicates depends just as much on scientific considerations (e.g., quality of the design, strength of the theory in question) as it does statistical ones, see (114, 115) for discussions.

## Discussion

Our two cognitive attitudes, Book of Truths and Book of Conversations, are extreme points on a continuum that are useful for examining how researchers evaluate claims and interpret replication results. They are not intended to perfectly describe any single researcher, nor are they intended to represent ideals that should be adopted. If a researcher’s intention is to provide a robust and accurate answer for some empirical question, which may underlie a policy or action, we would say that they hold a Book of Truths attitude in that situation. Holding this attitude can be problematic when the underlying effects to be replicated are contingent upon unobserved heterogeneity (76), hidden moderators (42), fundamental measurement challenges (116, 117), or other issues relating to generalizability (85, 118). These issues may be the norm for some fields,^‖^ and such replications are better evaluated from a Book of Conversations attitude—where the results are intended to inform other scientists and improve developing theory—but should not be taken as definitive and/or suitable for forming the basis of policy or action and should not be communicated as such to other scientists or lay audiences.

Returning to the central aim of our discussion, we say that a replication decision is sound if there is alignment between the replication decision and the researcher’s values and cognitive attitude. For example, the decision to perform two or more randomized trials with preregistered protocols and carefully prespecified statistical analysis plans that aim for the assessment of the benefits and harms of a new vaccine via direct replications is well aligned with the value of mitigating harm and a Book of Truths attitude. Conversely, employing a metastudy design, with many treatment manipulations to explore potential confoundings, is well-aligned with a Book of Conversations attitude. To be clear, there are many ways for values, attitudes, and decisions to be aligned (or not). We are not offering a definitive statement on the topic. There is considerable room for differing views and argumentation. Our framework provides researchers a structured way to answer the question: “why was this replication carried out the way it was?”

To this end, we recommend that researchers answer the following four questions when writing up the results of a replication study for publication (Table 1): 1) *What nonepistemic values are related to, or impacted by, this replication?*; 2) *What epistemic values should be considered when evaluating evidence resulting from the replication?*; 3) *What cognitive attitudes do I hold about the replication?*; and 4) *Is there alignment between my replication decisions and my values and attitude?* Table 1 provides specific instances of these questions. This could take the form of a short paragraph in the write-up when describing methods and design.

**Table 1.** Four types of questions to answer when deciding whether and how to replicate

Question types	Some specific questions	Examples
*What nonepistemic values are related to, or impacted by, this replication?*	Is there a larger harm we are trying to mitigate for a population? How will broader audiences use the results from the replication?	The nonepistemic value of harm mitigation and accurate lay audience communication are important when considering a health treatment replication.
*What epistemic values should be considered when evaluating evidence resulting from the replication?*	Will the replication results be used to develop or test a theory?	The epistemic value of theory development is important when establishing robust phenomena, and can prompt researchers to utilize replication tools designed to eliminate publication bias, such as registered replication reports.
*What cognitive attitudes do I hold about the replication?*	Is the aim to establish a highly robust and replicable result (Book of Truths) or not (Book of Conversations)? How do I consider the claims of the original study?	The perspective of The Book of Conversations aligns well with conceptual replication studies, which aim to study the behavior of various independent and dependent variables.
*Is there alignment between my replication decisions and my values and attitudes?*	How does my experimental design connect to my values? Is this consistent with my cognitive attitude on the replication?	Multiple similar studies done by different teams (e.g., multiple clinical trials) can be useful for establishing a definitive result to mitigate harm, such as in evaluating a new vaccine.

Our discussions and conceptual model, see Fig. 1, build upon previous work examining replication practices (119–123) by making a clear distinction between epistemic and nonepistemic values. This is useful as these values can sometimes interact with one another. A nonepistemic value like transparency can directly impact epistemic values relating to empirical evidence, e.g., a scientist may be far more skeptical of a claim if important experimental details have not been reported in sufficient detail. We also extend prior work by considering how these values interact with the cognitive attitudes of scientists, that is, how scientists evaluate claims and what functions they think the peer-reviewed literature should serve. Taken together, these factors play a role in the efficacy of replication tools, including journal reform measures such as reproducibility audits, registered reports, or the awarding of preregistration badges. The dialog titled “The Misalignment of Incentives in Academic Publishing and Implications for Journal Reform,” published in this Special Feature, offers ideas for future-oriented journal reforms that align with the goals of science. Similarly, the perspective “Automating the Practice of Science—Opportunities, Challenges, and Implications” discusses how automated scientific practices may accelerate science and enhance transparency and reproducibility.

## Data Availability

There are no data underlying this work.

## Footnotes

^*^This is known as Miller’s law (19), please see (20) for a thoughtful review on this topic.

^†^In science, it is hardly possible to unequivocally speak of facts or of truths. Likewise, whether or not an effect exists is not always a binary question, i.e., treatment manipulations rarely produce population effects identically equal to zero, although they may be so small that they are negligible (21). In practical terms, we will use the terms “highly robust” and “replicable” when describing results from a Book of Truths perspective.

^‡^Generally speaking, waste due to researcher’s lost time and effort needlessly replicating a finding could be another type of harm to mitigate.

^§^We are referring to computational reproducibility, where rerunning the same code on the same data, as in the original study, yields identical results.

^¶^Delineating between core and auxiliary features has analogous challenges in statistical modeling, see Navarro (79) for a discussion.

^#^Registered reports can be a useful tool for combating this particular problem.

^‖^For example, see ref. 118, and the many commentaries, for a recent discussion of generalizability concerns as they pertain to the behavioral sciences.
